# Selections and Abstracts

**Published:** 1914-08

**Authors:** 


### SELECTIONS AND ABSTRACTS

### THE TREATMENT OF PLACENTA PRAEVIA.

By Edward P. Davis, M. D., Philadelphia.

In the advance which the science of obstetrics has made in the last fifteen years, this serious condition with others of like importance, has received new consideration, and some changes have been suggested in its treatment.

At present we recognize that septic infection is quite as important in causing the death of the mother as hemorrhage or shock. It has also been repeatedly demonstrated that placenta praevia causes such a vascular condition of the cervix and lower uterine segment that forcible dilatation or vaginal caesarian section exposes the patient to great danger from hemorrhage and infection. Furthermore, so grave a complication as placenta praevia demands hospital facilities and the skill of experienced hospital operators.

My experience leads me to agree with the conclusions just stated, and I desire, in addition, to present this subject to you from the following point of view: Logically, placenta praevia is a variety of ectopic gestation. The normal location of attachment of the impregnated ovum during its greatest period of development is the wall of the upper expulsive segment of the uterus near the orifice of the fallopian tube from which it emerged. When the impregnated ovum remains in the graafian follicle, the fallopian tube or the upper wall of the uterus, its growth inevitably causes rupture and hemorrhage. The danger of septic infection, however, is primarily inconsiderable. When the impregnated ovum attaches itself below its usual site of development, hemorrhage is likewise inevitable because pregnancy must be terminated with dilatation of the cervix, and dilatation inevitably produces separation and bleeding. The danger of infection is equally great because pathogenic bacteria are frequently present in the vagina, and repeated examination and manipulation must convey them to the placental site. Hence there are some conditions in favor of the patient with the upper variety of ectopic gestation as against the patient who has the low variety, or placenta praevia. From this point of view, that method of treatment will be most successful which controls hemorrhage with the minimum risk of infection.

Again, cases of placenta praevia may be, for purposes of treatment, divided into those in which hospital facilities are promptly obtained and those which are treated outside of hospital. In hospital cases, operations which the general practitioner cannot safely attempt may be undertaken with a fair chance of success. If ectopic pregnancy of the upper variety is of sufficient gravity to demand the services of a skilful operator, with hospital facilities if possible, ectopic gestation of the lower variety, or placenta praevia, is equally grave.

Let us consider first those cases which comes under the care of the general practitioner of medicine and which cannot be conveyed to hospital.

Two important factors must be kept in mind under these circumstances:

Any vaginal hemorrhage in a pregnant woman is a symptom of danger and must not be neglected. Because hemorrhage from placenta praevia is accompanied by no pain, it is frequently neglected by patients.

2. In dealing with these cases the danger of infection must be kept prominently in view. Vaginal examinations and manipulation must be as infrequent as possible, and the use of the tampon should be avoided.

Without minute classification, placenta praevia may be divided into those cases in which the os is not entirely covered by placenta and in which the membranes can be reached, and those cases in which the membranes cannot be reached. In the hands of the general practitioner and in private houses, the best treatment in the first class of cases is to rupture the membranes as extensively as possible, when dilatation permits this maneuver. Tonic doses of strychnine are then given and the patient is allowed to deliver herself, or, at least, is made to force the presenting part down to the vulva before artificial delivery is undertaken. In this way hemorrhage and infection will be reduced to a minimum, and although the risk to the child is great the mother's safety should be first considered.

In complete placenta praevia, in the hands of the general practitioner in a private house, the Braxton-Hicks method of bringing down the leg and breech of the child as a plug should give the mother the best chance. To accomplish this, anesthesia, partial or complete, may be required. The placenta must be perforated by one or two fingers, combined version performed, and the leg seized and drawn through the placenta. Only sufficient traction should be made to bring the breech firmly into the pelvic cavity and against the cervix and placenta. Under no circumstances should forcible and immediate extraction be practiced. The life of the child should be disregarded in the interests of the mother. If a constant tension on the child is desired, a noose of bandage should be slipped about the child's ankle and a moderate weight attached. The mother may then be stimulated as required, care being taken to avoid the use of ergot and pituitary extract. Strychnine, atropin and digitalin hypodermically, with salt solution injected into a vein or connective tissue, will be found especially useful. The mother demands constant observation until labor finally develops, and the fetus, if possible, is expelled spontaneously. In placenta praevia the practitioner must expect and be forewarned against post partum bleeding. The delivery of the child should be followed by the delivery of the placenta, irrigation of the uterus with a hot 1 per cent. dilution of liquor cresolis compositus or normal salt solution, and firm packing of the uterus, cervix and vagina with 10 per cent. iodoform gauze. If undue traction is avoided and spontaneous expulsion of the fetus secured, there will be no dangerous laceration of the cervix; but if undue violence has been employed the cervix may be severely torn, and severe or fatal hemorrhage may follow. Under these circumstances immediate suture of the cervix may be necessary.

In hospital practice, with the mother in good condition and the membranes available for rupture, the escape of the amniotic fluid may be followed by the introduction of a dilating bag through the rent in the membranes into the cavity of the amnion. This should be gradually distended to its fullest extent securing dilatation of the cervix with pressure on the placenta. When dilatation is sufficiently advanced the child may be delivered by forceps, in many cases advantageously, with a minimum risk to the mother and with a great reduction in fetal mortality.

When the membranes are not available for rupture, and when the cervix is resisting, because in a primipara it is poorly developed, or in a multipara has been altered by scar tissue from previous confinement, mother and child will obtain the best result by prompt resort to abdominal caesarian section. If the child is premature or dying or dead, from long-continued maternal hemorrhage, the placenta may be perforated by the fingers and combined version practiced and the child's body used as a plug to check maternal bleeding. Spontaneous delivery and uterine contraction must be secured in the manner already described.

If the child be living and in good condition, the cervix soft and dilatable, and the head presenting, with the patient in hospital, the obstetrician may elect to pierce the placenta and insert a dilating bag through it within the cavity of the amnion. This bag may be distended with antiseptic fluid, hemorrhage checked by pressure, and gradual dilatation of the cervix thus secured. With good dilatation the use of the forceps will sometimes secure a living child. In hospital practice, however, where the child is fully viable, mother and child will obtain the best results by abdominal caesarean section. At full term in hospital cases, whether the child be living or dead, the mother will obtain her best chance of rceovery by the same method. Our experience in abdominal caesarian section for placenta praevia comprises seventeen cases. In these the membranes were not available and the placenta practically covered the entire internal os. In some of these patients the child was dead or dying and the mother exsanguinated. All were at or near term.

The method of operation consisted in opening the abdomen, turning out the uterus, incising it and immediately removing its contents. It was interesting to observe how completely hemorrhage ceased when the uterus was emptied. The uterine cavity was then thoroughly irrigated with hot sterile salt solution and packed firmly with 10 per cent. iodoform gauze carried through the cervix into the vagina. The uterus and abdomen were closed in the usual manner. The vagina was sponged out with mercuric chloride solution and firmly tamponed with sterile or mercuric chloride gauze. During the operation the patient received intravenous saline transfusion, and strychnine, digitalin and atropin were given hypodermically. In exsanguinated cases the patient was kept in the Trendelenburg posture until reaction occurred, and external heat, rectal injection of stimulants and oxygen inhalation were used with success.

Of the seventeen patients three were especially exsanguinated through severe and repeated hemorrhages. All the mothers recovered. The maternal mortality was nil; the fetal mortality 40 per cent. There was no maternal morbidity except slow recovery incident to severe anemia. Infection did not develop.—Journal A. M. A.

250 South Twenty-First Street.

---

### THE IMPORTANCE OF UROLOGY IN WOMEN.*

By Henry Dawson Furniss, M. D., New York City.

A very large percentage of our gynecological cases complain of urinary disturbances which are independent or dependent upon diseases of the genital organs. And yet it is surprising to see how very little these have interested the average gynecologist.

Many feel that gynecology is a very limited specialty, and it certainly is if we are to exclude from it the diseases of the urinary system. For the best interests of our patients I think it incumbent upon every gynecologist to acquire a good working knowledge of the methods of diagnosis and therapy of urinary diseases.

Of all diseases I know of none in which we are better able, with our present diagnostic methods, to make an accurate and comprehensive diagnosis, than in diseases of the uropoetic system, and without such methods and depending upon symptoms alone, none in which the chances of error are greater. In few are the classical symptoms present, and exactly the same symptoms are present in many conditions that are entirely different. For instance, I have seen renal calculi which have been present for months and years and have caused no symptoms; on the other hand, I have observed cases of renal tuberculoses, stricture of the ureter, and floating kidney, which have prevented text-book symptoms of calculus colic.

Below I shall outline our usual procedure of examination.

First.—A careful history from which we can infer the probable nature of the trouble and determine in what direction the examination had best be conducted.

Urinanalysis.—Examination of the specimen of urine which the patient has brought with her, or better still, examination of a catheterized specimen taken under aseptic conditions. Besides the usual microscopic and chemical examinations, a search should be made for tubercle bacilli in all cases.

I know of a pathologist in this city who has made several diagnoses of tuberculosis, where the physician sending the specimen had not suspected it and had not requested that tubercle bacilli be looked for.

In women the finding of tubercle bacilli in the urine is a practical certainty that at least one kidney is involved. A culture should always be taken, whether or no there is pus.

A careful pelvic and abdominal examination should now be made, to determine the mobility, the size, and the sensitiveness of the kidneys and any abnormalities of the genital organs. We should endeavor to palpate the ureters, for frequently low stones and thickened tuberculous ureters can be felt. In some cases of very severe tubecuulous cystitis it is impossible to determine with the cystoscope the involved side, and in these the thickened ureter alone furnishes a clue to the situation.

Of the many valuable functional tests, for practical purposes I find two sufficient: phenolsulfonephthalein test either intravenously or intramuscularly, and indigo-carmine intravenously. By the first we determine the total functional activity of the two kidneys; if the elimination is normal we can infer that at least one kidney is doing its work, if it is low, that neither kidney is efficient. The indigo-carmine test will be referred to below.

With the phenolsulfonephthalein test I have detected kidney insufficiency in cases where the usual methods of examination showed practically normal conditions. This test is of value not only in surgical but in medical cases, and it is of much importance in determining the advisability of doing operations that are more or less elective. On several occasions the constant low output has made me abandon operative interference, and the wisdom of this decision has been borne out by seeing the patients die within a short time.

All patients should be radiographed, for the findings are always instructive whether positive or negative, and at times surprising. Recently I sent a case to a orthopedist for chronic backache and the X-ray showed shadows suspicious of stone; this same orthopedist referred to me a case for kidney conditions, and the radiograph showed involvement of the sacro-iliac joint.

Should suspicion of stone be found the patient should again be radiographed, after the introduction of an X-ray catheter or injection into the kidney pelvis or ureter of argyrol or collargol, for in most ureteric stone cases there is dilatation of the ureter above the stone. These radiographs had best be made stereoscopically to make sure of the relation of the shadow to the ureter.

With the cystoscope and endoscope the whole of the bladder can be seen, and in most cases diagnosis of vesical conditions made.

The greatest difficulty is in interpreting the changes seen in and round the ureteric orifices. In women about half of bladder disturbances are due to chronic urethritis, and unless the urethra is inspected through the Kelly endoscope, these are overlooked and the case treated in vain for other conditions.

At the time of the cystoscopy, if sufficient has not been found to account for the symptoms, 10 c. c. of a 3-10 1% solution of indigo-camine is injected intravenously and the elimination from the two ureters observed through a simple examining cystoscope. This normally begins in from 2 to 6 minutes, the average time being 3½ minutes. In this way the determination of the better kidney is made by the relative time of the beginning elimination, and I think it better than by the phenolsulfonephthalein method which requires ureteral catheterization. Occasionally in difficult cases we use this method to locate a ureter or the presence of supernumerary ureters.

The ureteral catheter is a very valuable instrument, though one used often ill-advisedly. I think the best urologist is one who finds the least necessity for the ureteral catheter. For example, it is far better, if possible, to determine by simple cystoscopy and the observation of the ureteral orifices the presence of a tuberculous kidney, than to attempt to catheterize a diseased ureter. We gain no additional information by such a procedure and traumatism is deleterious.

The wax tip catheter is valuable in detecting the presence of such stones as show no shadows in the radiographs, and we occasionally use it.

One of our most valuable diagnostic aids is pyelography. It consists in radiographing the kidney, pelvis and ureter after injection into them of substances resistant to the passage of X-rays. Collargol and argyrol are the substances most used.

Abnormalities of form, size, and position as are found in hydrophorosis, floating kidney, stricture of the ureter, tumors encroaching upon the renal pelvis are graphically pictured.

When all other methods of diagnosis have failed to reveal the condition pyelography has often been our salvation. It is highly useful in strictures that are passed with little resistance and no recognition in the early stage of hydronephrosis.

A well known method of diagnosis and one used infrequently and usually as a last resort is tuberculin, but only in suspicious cases where there has been failure to discover the tubercle bacilli, either in smears or by guinea pig inoculations. A general reaction signifies only that tuberculosis is present and it is necessary to obtain a distinct focal reaction to localize the seat of the disease. There are more chances of discovering tubercle bacilli during and after such a reaction.

For lack of time I shall speak of only two therapeutic uses of the cystoscope: one is fulguration treatment of papillomata of the bladder, which has given excellent results with very little disturbance and hardly any damage to the bladder or urethra. The other is the ease of removal of foreign bodies through the endoscope. In this way I have taken out three hairpins and one gauze sponge, with no damage to any of the patients, though one of the hairpin cases was a five year old girl.

In cases of uretero vaginal fistula the opensing is so small and so hidden away as to be detected with great difficulty. In such cases I have found the most satisfactory method is to inject indigo-carmine intravenously and tampon the vagina with moist cotton. The patient is then cystoscoped and the elimination noted. If the severance of the ureter is complete that ureter will fail to eliminate the dye. If the severance is only incomplete the dye is eliminated in decreased amount. In this way we determine the side on which the ureter is injured. After ten minutes the cotton is removed from vagina and the blue stained portion will show us the location of the fistulous opening.

A point to be remembered is that in cases of long standing the renal function is impaired by infection and back pressure due to the contracted opening, and consequently the indigo-carmine will not be eliminated so strongly as by the normal kidney. I have not attempted to give a comprehensive and detailed account of the methods of examination, but only to outline our methods of procedure and to demonstrate the importance of urology in women.—N. Y. State Medical Journal.

---

### SIXTEENTH ANNUAL MEETING AMERICAN PROCTOLOGIC SOCIETY.

Held at Atlantic City, N. J., June 22 and 23, 1914.

President, Jos. M. Mathews, Louisville, Ky.; Vice-

President, Jas. A. McMillan, M. D., Detroit, Mich., in the chair.

Officers for the Ensuing Year.—President, Louis J. Krouse, M. D., Cincinnati, Ohio; Vice-President, Collier F. Martin, M. D., Philadelphia, Pa.; Secretary-Treasurer, Alfred J. Zobel, M. D., San Francisco, Cal.

Executive Council.—Jas. A. MacMillan, M. D., Detroit, Mich., Chairman; Louis J. Krouse, M. D., Cincinnati, Ohio; Lewis H. Adler, Jr., M. D., Philadelphia, Pa.; Alfred J. Zobel, M. D., San Francisco, Cal.

The place for meeting for 1915 will be San Francisco, Cal. Exact date and headquarters will be announced later.

The following were elected Associate Fellows of the Society:—Dr. Wm. H. Axtell, Exchange Block, Bellingham, Wash.; Dr. Rolla Camden, 915 Avenue of the Presidents, Washington, D. C.; Dr. Doscum C. McKenney, 1250 Main St., Buffalo, N. Y.

The following is an abstract of the principal papers read:

---

Extracts From the Reports on Proctologic Literature From March, 1913, to March, 1914. By Samuel T. Earle, M. D., of Baltimore, Md.

In Samuel T. Earle's review of Proctologic Literature from March, 1913, to March, 1914, he quotes from the following authors giving the salient points from each of their papers:

Percival P. Cole, M. B., Ch. B., F. R. C. S., England (British Medical Journal, Vol. 1, 1913, page 431); "The Intramural Spread of Rectal Carcinoma."

Robert A. Bachman, M. D., Newport, R. I., Surgeon U. S. Navy (Journal American Medical Association, Vol. L., 1913, page 1154); "A New Method for Hemorrhoids."

Jerome M. Lynch, M. D., New York City (The American Journal of Obstetrics and Diseases of Children, February, 1914, page 322); "Blocking the Sympathetic by a Method

Other than Spinal Anesthesia to prevent shock in the combined operation for Cancer of the Rectum, or Recto-Sigmoidal Juncture, with some Improvements and Modifications of Technic."

Charles R. Robins, M. D., Richmond, Va. (The Old Dominion Journal of Medicine and Surgery, May, 1913, Vol. XVI., page 236); "Sliding the Rectum in the Cure of Various Defects."

Granville S. Hanes, M. D., Louisville, Ky. (Kentucky Medical Journal, Vol. XI, June 25, 1913, page 516); "Anal Pruritus Treated by Operation; Report of Case."

Frederick H. Williams, M. D., Boston, Mass. (New York Medical Journal, Vol. XCVII, 1913, page 875); "Electicity in Rectal Diseases. A Neglected Resource in their Treatment."

T. F. Riggs, M. D., Pierre, S. D. (The St. Paul Medical Journal, Vol. XV, page 461); "Fistulo-in-Ano; Its Rational and Successful Treatment."

P. Lockhart Mummery, F. R. C. S., England (The Lancet, Vol. II, 1913, page 72); "Operation and After-Treatment of Fistula-in-Ano."

Harvey B. Stone, M. D., Baltimore, Md. (Annals of Surgery, Vol. LVIII, 1913, page 647); "Immediate and Late Results of the Whitehead Operation for Hemorrhoids."

Daniel Fisk Jones, M. D., Boston, Mass. (Boston Medical and Surgical Journal, Vol. CLXIX, page 707); "Carcinoma of the Rectum."

James W. Heslop, M. D., M. R. C. S., Newcastle-on-Tyne, England (The British Medical Journal, February 28, 1914, page 476); "Dissemination in Carcinoma of the Rectum."

---

### COCCYGODYNIA: A NEW METHOD OF TREATMENT BY INJECTIONS OF ALCOHOL.—By FRANK C. YEOMANS, A. B., M. D., OF NEW YORK CITY, N. Y.

The diagnosis is established by a thorough examination, both general and local. Local examination is made by inserting the index finger into the rectum and palpating the coccyx between it and the thumb outside. The soft parts intervening between the coccyx and anus are now compressed and the point of maximum tenderness is thus located, usually just beyond the tip of the coccyx. Proctoscopy rules out rectitis.

The prognosis hitherto has been better in the traumatic cases than in those of frank neuralgia or neuritis. The writer confidently predicts that the treatment proposed will render the latter equally amenable to treatment.

The writer proposes a treatment based on the suggestion of Schlosser in 1907, of injecting 70 to 80 per cent. alcohol in sensory nerves, thereby causing their degeneration as practiced in trifacial neuralgia.

The technique is simple and can be carried out in the office under strict aseptic precautions. The patient with empty bowel is placed on a table in the Sims' position and the skin about the coccyx painted with tincture of iodine. A 2 c. c. Luer or similar syringe is filled with 80 per cent. alcohol and armed with a two inch needle. The right index finger is now inserted into the rectum and the point of maximum tenderness is determined by counter pressure with the thumb outside. Maintaining the finger in the rectum to guard against puncture and as a guide, the needle is introduced through the midline directly to the painful spot, and 10 to 20 minims of solution are injected slowly.

The needle is withdrawn and its puncture sealed with collodion. The pain from the injection lasts a few minutes and is followed by a dull ache which may last a day or two. From three to five injections are usually required at intervals of about one week.

The writer reports seven cases, all women, treated from two months to four years ago. They required three, four or five injections each at intervals of about one week. Relief was prompt and complete and all the patients have remained well.

---

### THE TECHNIQUE OF THE PERINEAL OPERATION FOR CANCER OF THE RECTUM. By J. A. MacMillan, M. D., of Detroit, Mich.

In every case a preliminary colostotomy must be considered imperative. The colostotomy provides the only means of discovering whether a radical operation is justifiable or not, supplies physiologic rest for the affected part, and later provides for aseptic conditions in the surgical field.

After thorough divulsion a circular incision is made at the muco-cutaneous line and carried up to the lower surface of the levator ani. Most of the dissection can be done by the fingers. It is not necessary to destroy the external sphincter. This step of the operation exposes a circular area of the levator ani about an inch and a half wide. Before proceeding further the hemorrhage should be controlled and the location of the affected glands determined.

The next step of the operation includes the division of the levator ani and the removal of lymphatic glands.

The peritoneum may be entered anteriorly and separated laterally which will leave the mesosigmoid as the only attachment of the bowel. This should be divided as far from its colonic attachment as possible in order to secure the retention of a good vascular supply for the proximal end of the bowel after the excision.

When the gut can be drawn down sufficiently to permit the excision of the affected portion and the attachment of the lower edge of the mucous membrane to the skin, excision is done and the sutures placed. Free drainage is necessary.

The colostotomy is not closed until the patient has been up and about for several weeks.

### MYASTHENIA GASTRO-INTESTINALIS. By V. Lee Fitzgerald, M. D., of Providence, R. I.

By the term "myasthenia gastro-intestinalis," is understood a weakness of the muscles of the abdomen, stomach, intestines, and their supporting ligaments, with a consequent downward displacement of any or all of the viscera.

Many patients suffering from myasthenia in its different forms are in danger of having suspensory or other operations performed upon them, whereas the intestinal stasis can be entirely removed by medical measures and the baneful effects of the underlying ptosis entirely removed.

The general aim in the treatment is the relief of the stasis, and the restoration of the prolapsed viscera to as near their normal position as possible.

The success in the treatment of these patients depends not only upon the relief of stasis, but also upon the patient's active and persistent co-operation.

For the past two years the writer has been treating cases of myasthenia as follows: The patient is given a thorough examination, including that of the gastric contents, urine, and faces. In case of myasthenia of the stomach with dilatation and prolapse the patient is put to bed and fed through a duodenal tube six or seven times a day, depending upon the amount of food needed to nourish the patient. This gives the stomach a complete rest, and its comes up into normal, or nearly normal, position, in from ten days to two weeks.

---

### FURTHER OBSERVATIONS ON PRURITUS ANI: ITS PROBABLE ETIOLOGICAL FACTOR; RESULTS OF TREATMENT. (A Fourth Report, Based on Results of Original Research.) By Dwight H. Murray, M. D., Syracuse, New York.

In this report on the fourth year's work of original research on pruritus ani, the author finds there is not much more to give to the profession beyond the confirmation of the work of previous years. He has yet no reason to doubt his claim for the infection theory of pruritus ani.

Twenty new cases have been examined during the past year. In all but two of these streptococcus fecalis has been demonstrated.

It has been found that occasionally the bacterial growth seems to be so lacking in strength that it is difficult to obtain an autogenous vaccine. It is not known why this is so unless it is owing to the very low grade inflammation produced by germs not so active as those found in many other infections.

During this year two cases were treated by other physicians who tried to follow his technique, but in neither case was improvement manifest notwithstanding that streptococci were found present by the author's bacteriologist and although the same quality of vaccines were used. With the consent of their physician the author took up the treatment. Improvement was marked. The only difference in the technique that he could discover was that the others injected the vaccine deep into the muscle instead of directly into the skin or immediately beneath it.

During the past year the author has had additional proof that the itching does not extend appreciably above the white line of Hilton. He has also had continued confirmation of his previous statement that the moisture found upon the parts is not a discharge from the rectum.

This year's work again shows that other rectal diseases are not present regularly with pruritus ani, and the belief is confirmed that they are coincidental instead of etiological.

No unfavorable sequelae arose from the vaccine injections. There is now no hesitation in running the dose up to two billion or more dead bacteria. One injection resulted in formation of a jelly-like material in the tissue but this was absorbed. Some time ago a similar swelling was opened and found to be sterile, and no trouble has resulted.

### A REPORT OF CASES OF PRURITUS ANI TREATED WITH CARNOTITE. By Samuel T. Earle, M. D., of Baltimore, Md.

Carnotite, a radio-active mineral, was used in the treatment of eight cases of pruritus ani and was found to be a very satisfactory palliative remedy.

---

### TREATMENT OF AMEBIC DYSENTERY BY EMETINE HYDROCHLORIDE. By Alfred J. Zobel, M. D., of San Francisco, Cal.

The writer gives a brief culling from the literature on the emetine treatment of amebic dysentery, and also a few words relative to the drug itself.

He states that in emetine hydrochloride we have a reliable, non-toxic drug possessing a definite specific action; which may be administered hypodermically, and yet which will permit of a sufficient dose being given without causing any depression, nausea, vomiting, or local reaction.

He reports two interesting cases in which the disease was present in one individual for ten, and in the other for fourteen years. Under the influence of emetine, within two or three days amebae, blood, mucous, froth, and foul odor disappeared from the dejections and their number greatly decreased; the racking tenesmus, bearing down feeling in the rectum, the colic, and the abdominal tension, discomfort, and gurgling absolutely ceased.

Proctoscopic examinations revealed the favorable influence of the drug upon the amebic ulcerations. No amebicidal irrigations were employed.

He further reports other cases seen by him in consultation which demonstrate most forcibly the necessity for a proctoscopic examination of the bowel and a microscopic examination of the feces in every instance where a diarrhoea lasts longer than a week, even though the patient has never lived in nor visited a locality where the disease is known to exist.

He advises that emetine should be given for at least three or four months at intervals before the patient should be considered free from the possibility of a recurrence, even though he is clinically cured and the amebae cannot be longer found in the stools.

---

AMEBIC DYSENTERY AND ITS TREATMENT. By Dr. Wm. M. Beach, of Pittsburg, Pa.

The writer of this paper states that:

(1) Amebic dysentery in the early stages may be cured with emetine. (2) In cases somewhat advanced emetine is efficacious and at least clinically curative. (3) The use of the duodenal tube, through which to introduce solutions of emetine to any portion of the intestinal tract, should receive trial and consideration. (4) For rapid cure, and control, cecostomy or appendicostomy is the best measure in advanced and chronic cases. (5) Direct irrigation from above is superior to rectal injections, in that it is less painful and more thorough. (6) The appendix should be removed in most cases of amebic dysentery. (7) The so-called specific emetine can be easily applied in weak solutions.

---

THE PATHOLOGIC SIGMOID COLON AND ITS SURGERY. By L. J. Hirschman, M. D., of Detroit, Michigan.

Studies with the fluoroscope and the sigmoidoscope have shown that true prolapse and invagination of the sigmoid colon into the rectum is not an uncommon condition. The author advocates shortening the mesentery of the sigmoid by attaching the mesentery of the invaginated or prolapsed portion to the root of the mesentery of the descending colon.

In a number of cases of obstruction to normal defecation, this obstruction will be found in women who give a history of a disturbed puerperium. Radiographic studies of these patients who give a history of chronic obstipation accompanied by pain and marked tenderness in the left lower abdominal quadrant and the region of the womb and broad ligaments, more often the left, show the presence of adhesions which angulate, displace or bind down the sigmoid. The cure of this condition involves the relieving of the adhesions and the covering of raw areas with omental, epiploic or mesenteric grafts, or the excision or short-circuiting of the sigmoid. Another class of adhesions of the sigmoid seriously obstructing defecation is caused by adhesions to the abdominal wound following laparotomy.

Hypertrophy or redundancy of the sigmoid colon is another pathological condition which has not infrequently been met with. When the walls of the bowel contain a large proportion of unyielding fibrous tissue, short-circuiting is insufficient and excision is insufficient and excision is indicated.

In malignant growths of the sigmoid colon, excision with immediate anastomosis is the ideal indication.

When inoperable it is the author's practice to always make the colostomy in the median line. This is done for the following reasons: First, the median incision is the best for exploratory purposes. Second, one has the choice of any part of the colon in the making of the colostomy. Third, one gets just as good adhesion and union, with no more liability to hernia, as in the side. Fourth, the patient is better able to cleanse and dress the colostomy in the median line. Fifth, it takes the colostomy opening away from the neighborhood of the iliac crests, and allows of the better fitting of retention apparatus and colostomy shields. Sixth, control of the median colostomy is just as satisfactory as the lateral.

The author has found no difficulty in securing colostomy control by using a small rubber catheter in the mesenteric opening beneath the spur and encircling the upper limb of the colostomy with the catheter, drawing it just snug enough that the mucous surfaces appose. The catheter is held in this position by a seraphine snap and is released by the patient when he wishes to defecate or expel flatus.

### MYXORRHEA COLI—MYXORRHEA MEMBRANACEA AND M. COLICA (MEMBRANOUS ENTERITIS—MUCOUS COLIC). By Dr. S. G. Grant, of New York City, N. Y.

The essayist explained that myxorrhea coli was a symptom complex characterized by constipation, abdominal pain, uneasiness or soreness and the periodic evacuation of jelly-like strips or casts of tenacious mucous, on the one hand, or colic on the other, and suggested that all mucous discharges be designated as myxorrhea coli with which understanding the former is called myxorrhea membranacea and the latter myxorrhea colica. The writer conceded that either type of myxorrhea coli may be secondary to neurogenic disturbances but strongly maintained that myxorrhea membranacea and myxorrhea colica are frequently produced by many other conditions and disease, medical and surgical, several of which may be factors in the same case. He had often known these conditions to be caused by psychic, neurogenic, gastrogenic and enterogenic disturbances, adenoidism, thyroid disease, impaired metabolism, abnormal menstruation, affections of the heart, liver and pancreas, inflammatory and ulcerative lesions (colitis), helminths, foreign bodies, prolonged or irritating colonoclysis, various lesions which induce chronic intestinal obstruction and led to coprostasis and autointoxication and other ailments which cause the hypersecretion or retention of mucus. The writer had observed patients who suffered at first from myxorrhea membranacea and later myxorrhea colica where the mucus became inspisated, irritating and excited enterospasm.

The writer maintained that the diagnosis was easy in uncomplicated cases and that myxorrhea membranacea could be recognized by its symptom complex, obstinate constipation, uneasiness and soreness or pain in the lower left abdominal quadrant and the periodic discharge of strips, casts, or jelly-like masess of mucus, and that where subsequent to these manifestations and in the absence of signs pointing to intestinal obstruction from other causes colic suddenly supervenes, one is justified in making a diagnosis of myxorrhea colica.

The essayist discountenanced a routine treatment in these cases and advised holding curative measures in the abeyance until the acute symptoms subsided.

The removal or correction of kinks, twists, strictures, invaginations, adhesions, pericolic membranes and other lesions obstructing the bowel or causing stasis, effected a cure in many of the writer's cases and he rarely found the bowel sufficiently incapacitated to require resection, exclusion, or the establishment of an artificial anus.

In conclusion, the writer stated that myxorrhea membranacea and myxorrhea colica were common affections and more frequently responded to surgical treatment than the literature of the subject would indicate.

---

### PERI-RECTAL GUMMA: REPORT OF TWO CASES.

By Alois B. Graham, M. D., of Indianapolis, Ind.

The subject peri-rectal gumma owes a great deal of its interest to its rarity. The author reports two cases which are rather unique. They were seen within twenty-four hours of each other, and both presented a typical peri-rectal gumma, in that no other lesion of any kind could be detected in the rectum of either patient.

The author's conclusions are that peri-rectal gummata are rare. The two cases reported are unique and of interest in that both were typical examples of peri-rectal gummata. In both cases the gumma was seen in its early or vascular phase. In one case it appeared 23 years after the initial lesion; in the other it appeared three years following the syphilitic infection. Both gummata were painless to palpation and fluctuation was detected in both. An error of diagnosis in one case was responsible for the incision and subsequent suppuration which followed. In the other case no incision was made and suppuration did not occur. No demonstrable rectal lesion could be discovered in either case. The induration in both cases disappeared rapidly under antisyphilitic medication. No fistula resulted in either case.

---

### ANAL AND RECTAL GROWTHS OF BENIGN OR DOUBTFUL CHARACTER. By Dr. T. Chittenden Hill, of Boston, Mass.

Hill states that in a series of 3,000 rectal cases previously reported there were 49 benign and 76 malignant growths of the rectum. The large majority of these tumors were characteristic and the differential diagnosis was easily made. A few malignant growths seen in an early stage, and some unusual benign types associated with ulceration, were of such a nature that the exact diagnosis was not easily determined.

The writer emphasized the fact that the operative measures to be employed differ radically in each of these conditions. An excision of the rectum is necessary for the malignant cases, a simple local excision is all that is required for the benign growths, where an incisio nand drainage will suffice for the abscesses and fistulae. Therefore, a doubtful case cannot be treated as a breast case in which a complete amputation for a benign growth may be justified. In the case of the rectum there is not alone mutilation but a high mortality and a serious impairment of function as well to be considered. Furthermore, the removal of a specimen of a suspected tumor is not now approved and this complicates the problem still more.

The histories of several cases which illustrate the doubtful nature of some border line conditions occasionally found in the rectum are cited. They tend to show that aside from benign growths some of which have many of the characteristics of malignancy, there are certain abscesses which develop in the loose cellular tissue of the rectro-rectal and pelvi-rectal spaces which are even more suspicious. These indurated, irregular swellings bulging into the rectal ampullae at first resemble very closely the sensation imparted to the finger in malignancy. A little later they become soft and fluctuation is perceptible when all doubt as to their nature is removed. The sinus from an old fistulae occupying these same spaces is apt to be much more perplexing than an abscess. As the slow process goes on the rectal wall is crowded into the lumen of the bowel and assumes an irregular, indurated outline which is very suggestive of cancer. Other conditions of similar doubtful cahracter such as gummatous growths and tubercular ulceration are also discussed.

---

RETRORECTAL INFECTIONS. By Collier F. Martin, M. D., of Philadelphia, Pa.

Martin reviews the histories of sixty-seven cases. In addition to the infection of the retrorectal space many of the cases also had involved the pelvirectal and ischiorectal spaces. Some of the more chronic cases were complicated with stricture of the rectum and multiple fistulae.

Eighty-five per cent. of the infections occurred in males. External traumatism was not a factor in this series of cases. The author holds that most of these infections originate from internal traumatism, associated with some condition which lowers the resistance of the individual to pyogenic infection.

Pulmonary tuberculosis appears to be the most constant factor in thus lowering the resistance. Twenty-one per cent. died from tuberculosis at varying periods, either after examination or operation.

Forty-three per cent. of the cases are noted as having pulmonary tuberculosis more or less advanced.

Of the fifty-five cases operated upon, thirty-three were cured. These present sixty per cent. of the operative cases, or nearly fifty per cent. of the total number examined.

In nearly half of the cases the original abscesses had opened posteriorly, either between the sphincters or at the anorectal line. Pain was not a prominent symptom.

The methods of incision applicable to the various complicating conditions are briefly outlined.

The author lays great stress upon the seriousness of these infections, and upon the necessity of the prolonged watchful after-treatment.

While the prognosis as to both complete recovery of the local condition and the general health, as well as to the preservation of the sphincter control, should be guarded, careful after-treatment and prolonged observation will result in saving a large proportion of these really serious cases.

An abbreviated history of the findings in the entire sixty-seven cases is given.

---

HEMORRHOIDS: THEIR TREATMENT. By Dr. J. Rawson Pennington, of Chicago, Ill.

Dr. Pennington states that clinically hemorrhoids should be classified:

(1) According to their location.

(2) According to their structure.

According to their structure they are divided into, (a) those containing fluid blood, (b) those containing clotted blood, (c) those containing both fluid and clotted blood, and (d) those consisting of "skin tabs" or folds of skin.

Most hemorrhoidal cases can be operated on under some form of local anesthesia. He operates on 90% of his cases by blocking the field of operation. The cocaine is usually employed in the strength of from ¼ to ½ of 1%. The quinine and urea in from ¼ of 1% to 1% solution. Sometimes he combines the solutions, the cocaine being used for its immediate effect and the quinine and urea for prolonging the anesthesia.

During the last 20 years he has given a fair trial to a number of methods advocated which promised a reasonably good result, including the ligature, the clamp and cautery, Whitehead, injection, suturing and other methods which unite tissue in mass, and has come very definitely to the conclusion that by far the best way of treating this condition is by the excision or enucleation method.

The operative procedure should have for its object the removal of the cause of the tumefaction. The treatment for each type of hemorrhoid should be practically the same. This should consist in removing an ellipse from the tumor-like formation and in the case of the thrombotic pile turning out the clot, and in that of the internal variety the varicosity and allowing the blood to escape, and in the fleshy pile of dissecting out the excess of tissue.

---

### HYPERPLASTIC TUBERCULOSIS OF THE COLON.

By J. M. Frankenburger, M. D., of Kansas City, Mo.

The writer declared that this form of tuberculosis of the intestine differs from other forms of intestinal tuberculosis, inasmuch as it is amenable to operative interference. It is generally a local and primary lesion and is characterized by the formation of tumor masses composed of fibrous and tuberculous granulation tissue in the walls of the bowel. Primarily there is no involvement of the mucous membrane, but on account of the narrowing of the gut the irritation caused by the passage of feces may produce ulceration.

Symptoms are slight, constipation and diarrhea sometimes alternating. Later the symptoms are those of gradually increasing intestinal obstruction. Differential diagnosis is between sarcoma, carcinoma, syphilis, and chronic appendicitis with adhesions.

Treatment is purely surgical. If possible the whole growth should be removed, but failing in this a short circuiting operation should be performed to relieve the obstruction.

Two cases are reported with successful operations.

---

### PSEUDO-INTESTINAL STASIS AND REAL INTESTINAL STASIS, DEMONSTRATED ROENTGENOLOGICALLY.

By Arthur F. Holding, M. D., of New York City, N. Y.

Attention is called to many anomalies of visceral position and progress of the bismuth meal that have been interpreted as pathologic, and which are really physiologic or anatomic anomalies and completely compatible with health, laying especial stress upon the fact that the ileum enters the caecum normally at an angle, and unless associated with proximal distension, a diagnosis of Lane's kink is not justified.

He emphasized the point that delayed progress of the bismuth meal is not significant of obstruction unless it is more than 6 hours behind the normal schedule and associated with marked distension of the viscus proximal to the locus of obstruction. Proximal distension with obstruction to the bismuth column are the two cardinal diagnostic points of real intestinal stasis. Intestinal obstruction, due to tumors, is much easier to diagnose than intestinal stasis, because the defect in the bismuth shadow made by the tumor is more definite than that made by adhesions, veils, or membranes.

---

LOCAL TREATMENT OF ANAL FISSURE. By Jas. A. Duncan, M. D., of Toledo, Ohio.

The writer describes a treatment for anal fissure which he has employed successfully for the past thirteen years. The fissure is brought into view by separating the folds, and the surface is lightly curetted, then thoroughly dried, and a drop of collodion applied. This takes only a moment or so. A recent ulceration requires but a single application. A sharp stinging pain lasting for only a few minutes is caused, and then the patient is left perfectly comfortable.

---

SOME UNUSUAL PHASES OF SIGMOIDOSCOPY. By Ralph W. Jackson, M. D., of Fall River, Mass.

The diagnostic value of the sigmoidoscope has been the topic of much writing, and is increasingly appreciated by hospitals, but much less so by the profession and insufficiently in medical teaching. Explicit statements of its considerable therapeutic uses are not found in German, American or English literature. The instrument enhances the extent and accuracy of recto-sigmoidal therapeutics, and specifically it facilitates the use of certain other instruments, topical applications, the relief of high impaction, and the treatment of stricture and many other lesions. Serious trauma from the sigmoidoscope is more liable to happen than some authorities admit, as illustrated by three cases of intestinal perforation cited from the German. Two personal cases are detailed, where the patients were in serious condition from occlusion of the bowel, but were relieved and saved by sigmoidoscopy done with diagnostic intent only. Pelvic visceroptosis, hypermobility of the sigmoid, and the fixed and open rectal ampulla beneath predispose to invaginations and angulations which are fairly frequent in mild and chronic form, and are potentially dangerous as a source of acute obstruction. Sigmoidoscopy, properly conducted, empties the pelvis by gravity (due to the position assumed) by intelligent introduction of the instrument and by the air pressure admitted through it, and therefore tends to undo such intestinal malpositions. The occlusion in the two cases related was unexpectedly relieved, and doubtless in this way. Greater prevalence in the use of the sigmoidoscope would bring to light a field for deliberate therapeutic use of the instrument along these lines.

---

CRUDE AND CARELESS DIAGNOSTIC METHODS, AND RESULTS OF SAME, IN SOME RECTO-COLONIC CONDITIONS. By Jno. L. Jelks, M. D., of Memphis, Tenn.

The author criticizes the busy doctor and surgeon who too hastily yields to a conclusion and treats recto-colonic diseases without sufficient investigation to warrant or obtain a correct diagnosis.

Reference is made to cases operated on for appendicitis, which disease may be an extension and inflammation originating in the rectum or colon.

Cases are cited to show the frequency and at all times the liability of mistaking a condition for an infection or ulceration of the colon, specific in character, when a coloptosis or pericolic membranes, or both, were the true etiologic factors. Stress is laid on the importance of urinalysis, microscopic examinations and the X-ray in recto-colonic cases.

A harder nodular calcareous degeneration of the outer zone of the mamma has been observed as a sequence of coloptosis and defective drainage. In another case, in which was found a cecum cradled in pericolic membranes, and a coloptosis, a duodenal ulcer was diagnosticated. In this case the urinalysis, the history, and general toxic appearance of the patient, pointed to true etiology.

Case reports are given in which diarrhea was the dominant symptom, though impaction, pericolic membranes, and ptosis were the true etiology.

The author calls attention to his prior reference to, and work of establishing the importance of conserving the ilio-cecal valve; also to the syphonage of a ptosed colon after short circuiting operations, which he accomplished by a second anastomosis between the blind colon and the sigmoid or rectum below the first anastomosis.

Importance is claimed for a microscopic examination of the intestinal contents of patients who suffer from attacks of appendicitis, and of the contents of the removed appendix; and the author insists that in the event that pathogenic amebae are found appendico-cecostomy should be performed instead of appendectomy.

The author refers to his observation of quite marked congestion of blood in the visceral vessels themselves in these cases of ptosis and defective intestinal drainage.

The author refers to the frequency with which he encounters cases of inoperable cancers of the rectum and intestines, the neglect of which is most often due to the fear of examination of those suffering with symptoms in the regions referred to.

To Be Continued.)

